# Supplementary material for: Beyond partisan filters: Can underreported news reduce issue polarization?
Source: PLoS One. 2024 Feb 16;19(2):e0297808. doi: 10.1371/journal.pone.0297808 (PMC10871475; doi:10.1371/journal.pone.0297808)
Supplement: S1 Appendix — (PDF) [file pone.0297808.s001.pdf]

Online Appendix for: Beyond Partisan Filters: Can Underreported News Reduce Issue Polarization?

Curtis Bram*

Contents

1 Ethics & participant recruitment **1**

2 AAPOR Items for immediate disclosure **2**

3 Newsletter stories and flowchart **3**

4 Measured variables **7**

5 Information on sample **9**
5.1 Descriptive data on sample 9
5.2 Balance check 11
5.3 Descriptive evaluations of the Blindspot Report 12

6 Disaggregating treatment effects across issues **13**
6.1 ATE results 13
6.2 Additional analysis for Cuban refugees issue 17

7 Analysis with demographic controls **20**

*Assistant Professor, The University of Texas at Dallas (curtis.bram@utdallas.edu).

1 Ethics & participant recruitment

All respondents completed an informed consent form before each survey they completed. This study was approved by Duke University's Institutional Review Board on July 8, 2021 (protocol number: 2021-0598). This study used no deception. On Friday July 23, a winner in the gift card lottery was randomly chosen and that day received the incentive as described.

Figure 1 shows the recruitment email sent to Blindspot Report subscribers. The email provides a link to the survey, highlights the incentive, and states that the survey attempts to understand how people think about political issues.

Figure 1: Recruitment email sent by Ground News

Hello Blindspot Report Readers,

Ground News is collaborating with Duke University on a research project that seeks to better understand how people think about political issues.

If you fill out a 5-minute survey and a brief follow-up survey on Wednesday, you'll be entered to win a **$500 Amazon gift card**.

FYI: Ground News is not being compensated for this study and all responses are completely confidential.

You can fill out the survey here.

Stay Grounded!

Harleen CEO - Ground News

Ground News

Duke UNIVERSITY

Want to change how you receive these emails?
You can update your preferences or unsubscribe

2 AAPOR Items for immediate disclosure

- Survey sponsor: Duke University provided funding for the study, Ground News facilitated recruitment and treatment delivery but was not involved in the analysis of these data
- Study dates: July 19 - 23, 2021
- Sampling frame: Subscribers to Ground News's "Blindspot report"
- Sample selection method: All respondents from sampling frame invited to complete survey
- Sample size: 1,234 respondents completed both survey waves
- Survey mode: Online
- Response rate: Exact response rate not available, about 115,000 people were emailed the invitation to take the survey
- Margin of error: not applicable
- Weighting: not applicable
- Questionnaire: relevant items included below
- Data access: available at https://doi.org/10.7910/DVN/QLF6MV

3 Newsletter stories and flowchart

Figures 2 through 5 each show one of the randomized stories that people could receive in their newsletter. Respondents randomly assigned to Newsletter A received Figures 2 and 3. Respondents randomly assigned to newsletter B received Figures 4 and 5.

Figure 2: **Example of a story about Cuban refugees that received most coverage from conservative-leaning sources, and which was also included as an experimental treatment. Reprinted from the original under a CC BY license, with permission from Ground News, original copyright 2023. Please note that the original figure included a photo that was omitted in this paper for copyright reasons.**

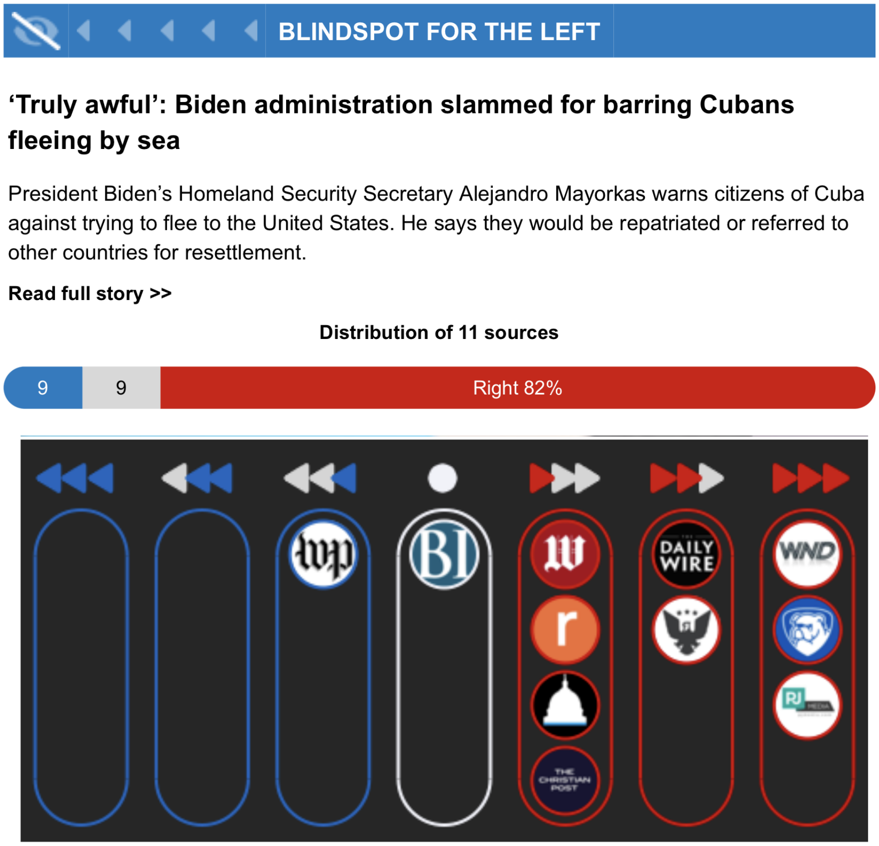

Figure 3: **Example of a story about the origins of Covid-19 that received most coverage from conservative-leaning sources, and which was also included as an experimental treatment. Reprinted from the original under a CC BY license, with permission from Ground News, original copyright 2023. Please note that the original figure included a photo that was omitted in this paper for copyright reasons.**

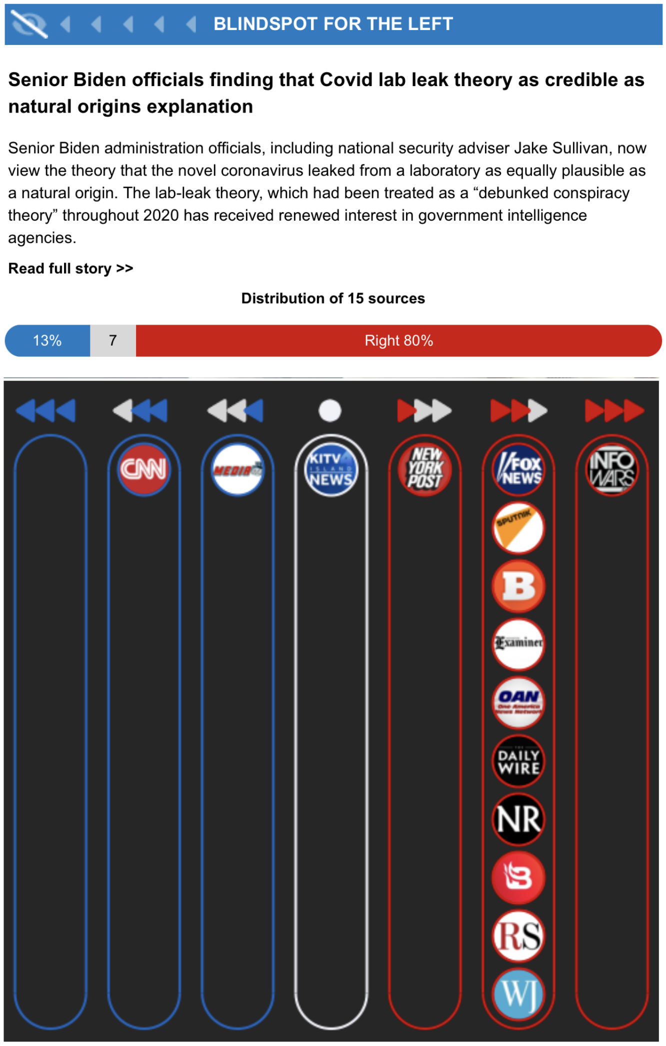

Figure 4: **Example of a story about Putin's plot to put Trump in the White House that received most coverage from liberal-leaning sources, and which was also included as an experimental treatment. Reprinted from the original under a CC BY license, with permission from Ground News, original copyright 2023. Please note that the original figure included a photo that was omitted in this paper for copyright reasons.**

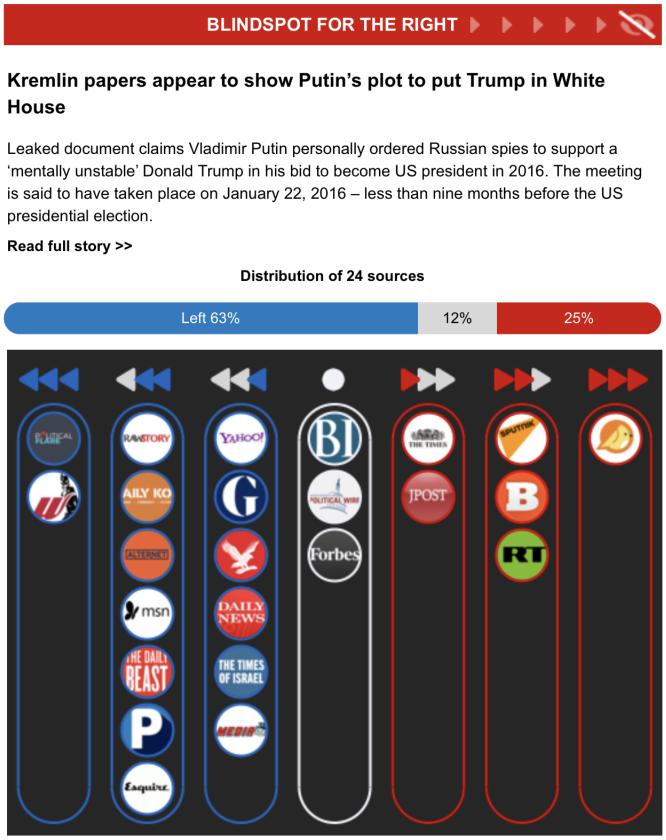

Figure 5: **Example of a story about vaccine misinformation that most coverage from liberal-leaning sourtces, and which was also included as an experimental treatment. Reprinted from the original under a CC BY license, with permission from Ground News, original copyright 2023. Please note that the original figure included a photo that was omitted in this paper for copyright reasons.**

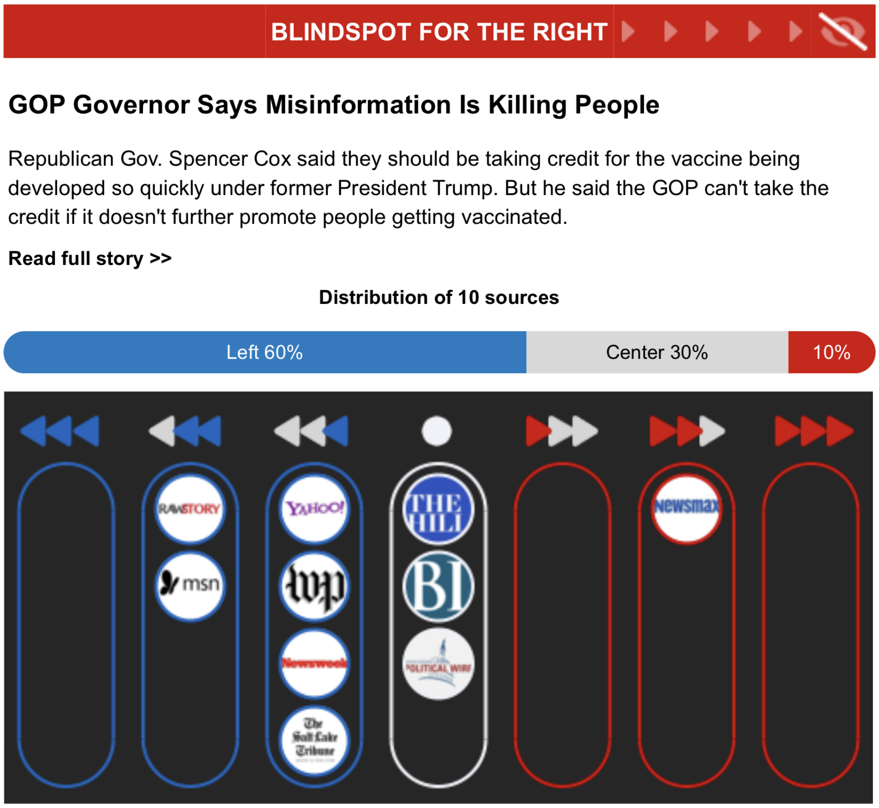

Figure 6: **Flowchart documenting the experimental design and timeline of surveys / newsletter emails**

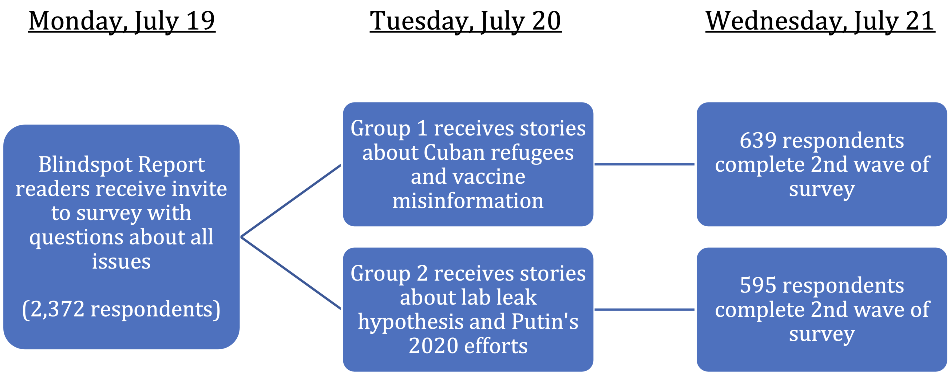

4 Measured variables

- Issue importance
  - Response options always: not important at all (0), a little important, somewhat important, very important, extremely important (1)
    * How important is the issue of Cuban refugees attempting to come to America by sea?
    * How important is the issue of Covid-19 vaccine misinformation?
    * How important is the issue of Russian intervention in American elections?
    * How important is the issue of investigating the origin of the Covid-19 pandemic?
- Issue positions
  - Response options always: strongly oppose (0), slightly oppose, neither favor nor oppose, slightly favor, strongly favor (1)
    * To what extent do you favor or oppose restricting entry for Cuban refugees attempting to come to America by sea?
    * To what extent do you favor or oppose more effort to address Covid-19 vaccine misinformation?
    * To what extent do you favor or oppose implementing additional sanctions on Russia for past intervention in American elections?
    * To what extent do you favor or oppose putting more pressure on China for answers about the origin of the Covid-19 pandemic?
- Issue polarization
  - Response options always: they strongly oppose (0), they slightly oppose, they neither favor nor oppose, they slightly favor, they strongly favor (1)
    * We'd like to find out what you think other peoples' positions are on restricting entry for Cuban refugees attempting to come to America by sea (Democrats / Republicans)
    * We'd like to find out what you think other peoples' positions are on more effort to address Covid-19 vaccine misinformation (Democrats / Republicans)

    * We'd like to find out what you think other peoples' positions are on the U.S. implementing additional sanctions on Russia for past intervention in American elections (Democrats / Republicans)
    * We'd like to find out what you think other peoples' positions are on putting more pressure on China for answers about the origin of the Covid-19 pandemic (Democrats / Republicans)

- Issue approval
  - Response options always: I strongly disapprove (0), I slightly approve, I neither approve nor disapprove, I slightly approve, I strongly approve (1)
    * To what extent do you approve or disapprove of each party's handling of the issue of Cuban refugees attempting to come to America by sea? (Democrats / Republicans)
    * To what extent do you approve or disapprove of each party's handling of the issue of Covid-19 vaccine misinformation? (Democrats / Republicans)
    * To what extent do you approve or disapprove of each party's handling of the issue of Russian intervention in American elections? (Democrats / Republicans)
    * To what extent do you approve or disapprove of each party's handling of the issue of investigating the origin of the Covid-19 pandemic? (Democrats / Republicans)

5 Information on sample

5.1 Descriptive data on sample

Table 1 provides summary statistics for the sample. Education is coded from 1 (did not complete high school) to 5 (graduate degree). The average respondent had completed a Bachelors degree. Democrat, Republican, Female, White, Black, Hispanic, and Asian are each indicators for identification with the category. One respondent claimed to be 121 years old, despite the oldest living person (at the time of this writing) being 118 years old. Table 2 provides summary statistics for those 642 respondents who Ground News confirms had opened the newsletter at about the time that the second wave of the survey launched. There do not appear to be meaningful differences between respondents who completed the survey and those who definitely opened the email at the time the recontact study was launched.

Table 1: Descriptive statistics for all respondents

Statistic	N	Mean	St. Dev.	Min	Max
Education	1,233	3.90	0.98	1	5
Democrat	1,234	0.26	0.44	0	1
Republican	1,234	0.18	0.38	0	1
Female	1,233	0.26	0.44	0	1
White	1,233	0.82	0.39	0	1
Black	1,233	0.02	0.13	0	1
Hispanic	1,233	0.04	0.20	0	1
Asian	1,233	0.05	0.21	0	1
Age	1,231	44.00	17.00	14	121
Non-US Respondent	1,234	0.25	0.44	0	1
Independent	1,234	0.31	0.46	0	1

Table 2: Descriptive statistics for respondents who had opened the newsletter at the time the second wave of the survey launched

Statistic	N	Mean	St. Dev.	Min	Max
Education	642	4.00	0.97	1	5
Democrat	642	0.27	0.45	0	1
Republican	642	0.18	0.38	0	1
Female	642	0.28	0.45	0	1
White	642	0.82	0.39	0	1
Black	642	0.02	0.13	0	1
Hispanic	642	0.04	0.20	0	1
Asian	642	0.04	0.20	0	1
Age	642	45.00	17.00	16	83
Non-US Respondent	642	0.23	0.42	0	1
Independent	642	0.31	0.46	0	1

5.2 Balance check

Table 3 is a balance check across experimental conditions. I find no statistically significant evidence of imbalances across conditions that correlates with measured characteristics. In total 639 people who received newsletter version A completed both waves of the survey, and 595 people who received newsletter version B completed the survey.

Table 3: Balance check

	Dependent variable:
	VersionDummy
Democrat	−0.04
	(0.04)
Republican	−0.05
	(0.04)
NonUS	0.003
	(0.04)
White	0.003
	(0.04)
Black	−0.15
	(0.12)
Hispanic	0.06
	(0.08)
age	−0.001
	(0.001)
Female	0.03
	(0.03)
Constant	0.59***
	(0.06)
Observations	1,231
R^2	0.01
Adjusted R^2	0.0004
Note:	*p<0.1; **p<0.05; ***p<0.01

5.3 Descriptive evaluations of the Blindspot Report

A grid question asking people to evaluate the Blindspot Report was included in the recontact wave after all other questions had been answered. The questions were as follows (all response options range from strongly disagree (0) to strongly agree (1):

- The Blindspot report helps me understand more about my own biases
- The Blindspot report helps me understand more about the biases of others.
- The Blindspot report makes me more understanding of views I disagree with
- The Blindspot report makes me more empathetic of those who hold views I disagree with.
- The Blindspot report has changed my mind about important political issues.

Results are that respondents felt that the Blindspot report helped them become aware of the biases of others, understand more about views they disagree with, understand their own biases, and become more empathetic of political opponents. Respondents are more equivocal (neither agree not disagree) about whether the Blindspot report changed caused them to change their mind about important political issues.

Table 4: Table S3: Respondent's evaluation of Blindspot Report

Statistic	N	Mean	St. Dev.	Min	Max
Empathetic	1,234	0.60	0.21	0.00	1.00
Change Mind	1,234	0.50	0.21	0.00	1.00
Others Biases	1,234	0.77	0.17	0.00	1.00
My Bias	1,234	0.72	0.18	0.00	1.00
Views Disagree	1,234	0.67	0.20	0.00	1.00

6 Disaggregating treatment effects across issues

6.1 ATE results

One way to look at this experiment is by looking at average treatment effects. That analysis is of limited use because one would never expect that receiving the content in the Blindspot report would have uniform effects. After all, a Democrat learning that President Biden is being more restrictive toward refugees than he or she expected is very different from a Republican learning the same thing. Still, these average results are the simplest way to analyze the results of this experiment.

Based on these results, no p-value was less than the Bonferroni-corrected significance threshold of 0.003125. This threshold was derived by adjusting the standard alpha level of 0.05 to account for the 16 potential tests - these result from each possible attitude-issue combination. Each of these individual hypotheses would need to meet this adjusted alpha level to reject the null hypothesis.

Table 5: ATE Results for Issue Importance

	Dependent variable:			
	Cuban Refugees	Vaccine Misinformation	Covid Origins	Russian Interference
	(1)	(2)	(3)	(4)
Cuban Treatment	0.005			
	(0.011)			
Vaccine Treatment		0.001		
		(0.010)		
WIV Treatment			−0.002	
			(0.009)	
Russia Treatment				0.016
				(0.010)
Constant	0.019^{**}	−0.002	−0.005	-0.021^{***}
	(0.008)	(0.007)	(0.006)	(0.007)
Observations	1,234	1,234	1,234	1,233
R^2	0.0002	0.00000	0.00004	0.002
Adjusted R^2	−0.001	−0.001	−0.001	0.001
Note:				*p<0.1; **p<0.05; ***p<0.01

Table 6: ATE Results for Personal Issue Position

	Dependent variable:			
	Cuban Refugees	Vaccine Misinformation	Covid Origins Position	Russian Interference
	(1)	(2)	(3)	(4)
Cuban Treatment	0.006			
	(0.018)			
Vaccine Treatment		0.003		
		(0.011)		
WIV Treatment			−0.010	
			(0.009)	
Russia Treatment				0.026^{**}
				(0.010)
Constant	0.007	0.005	-0.011^{*}	-0.014^{*}
	(0.013)	(0.008)	(0.006)	(0.007)
Observations	1,233	1,234	1,234	1,233
R^2	0.0001	0.0001	0.001	0.005
Adjusted R^2	−0.001	−0.001	0.0001	0.004
Note:				*p<0.1; **p<0.05; ***p<0.01

Table 7: ATE for Perceived Issue Polarization (measured as the difference between people's perceptions of Democrat and Republican positions on each issue.)

	Dependent variable:			
	Cuban Refugees	Vaccine Misinformation	Covid Origins	Russian Interference
	(1)	(2)	(3)	(4)
Cuban Treatment	−0.039**			
	(0.018)			
Vaccine Treatment		0.011		
		(0.013)		
WIV Treatment			−0.004	
			(0.012)	
Russia Treatment				0.029
				(0.020)
Constant	−0.039***	0.180***	0.170***	−0.008
	(0.013)	(0.009)	(0.008)	(0.014)
Observations	1,229	1,233	1,231	1,228
R^2	0.004	0.001	0.0001	0.002
Adjusted R^2	0.003	−0.0002	−0.001	0.001
Note:				*p<0.1; **p<0.05; ***p<0.01

Table 8: ATE for Issue Approval Difference (measured as the difference between approval ratings of Democrat and Republican politicians).

	Dependent variable:			
	Cuban Refugees	Vaccine Misinformation	Covid Origins	Russian Interference
	(1)	(2)	(3)	(4)
Cuban Treatment	−0.010			
	(0.016)			
Vaccine Treatment		−0.021		
		(0.016)		
WIV Treatment			−0.001	
			(0.017)	
Russia Treatment				0.005
				(0.017)
Constant	−0.006	0.007	0.036***	0.014
	(0.012)	(0.012)	(0.011)	(0.012)
Observations	1,227	1,233	1,232	1,229
R^2	0.0003	0.001	0.00000	0.0001
Adjusted R^2	−0.0005	0.0005	−0.001	−0.001
Note:				*p<0.1; **p<0.05; ***p<0.01

6.2 Additional analysis for Cuban refugees issue

Table 9: Change in issue polarization for Cuban issue

	Change in attitudes
Baseline	-0.04^{**}
	(0.01)
Democrat received blindspot	-0.08^{**}
	(0.03)
Independent received story	-0.02
	(0.03)
Non-U.S. respondent received blindspot	-0.04
	(0.03)
Republican received in-partisan	0.01
	(0.03)
R^2	0.01
Adj. R^2	0.01
Num. obs.	1229

$^{***}p < 0.001$; $^{**}p < 0.01$; $^{*}p < 0.05$

Table 10: Change in issue polarization on Cuban issue among only those who Ground News confirms opened the newsletter

	Change in attitudes
Baseline	-0.05^{*}
	(0.02)
Democrat received blindspot	-0.16^{***}
	(0.04)
Independent received story	-0.04
	(0.04)
Non-U.S. respondent received blindspot	-0.09^{*}
	(0.04)
Republican received in-partisan	-0.06
	(0.05)
R^2	0.03
Adj. R^2	0.02
Num. obs.	639

$^{***}p < 0.001$; $^{**}p < 0.01$; $^{*}p < 0.05$

Table 11: Change in people's issue positions for Cuban issue

	Change in attitudes
Baseline	-0.01
	(0.01)
Democrat received blindspot	-0.01
	(0.03)
Independent received story	-0.05
	(0.03)
Non-U.S. respondent received blindspot	-0.00
	(0.03)
Republican received in-partisan	0.07^{*}
	(0.03)
R^2	0.01
Adj. R^2	0.00
Num. obs.	1233

$^{***}p < 0.001$; $^{**}p < 0.01$; $^{*}p < 0.05$

Table 12: Change in people's issue positions for Cuban issue among those who Ground News confirms to have opened the newsletter

	Change in attitudes
Baseline	-0.01
	(0.02)
Democrat received blindspot	0.02
	(0.04)
Independent received story	-0.05
	(0.03)
Non-U.S. respondent received blindspot	-0.02
	(0.04)
Republican received in-partisan	0.08
	(0.05)
R^2	0.01
Adj. R^2	0.01
Num. obs.	641

$^{***}p < 0.001$; $^{**}p < 0.01$; $^{*}p < 0.05$

7 Analysis with demographic controls

	Issue polarization	Positions	Importance	Approval
(Intercept)	0.07*	−0.01	−0.02*	0.00
	[0.03; 0.11]	[−0.03; 0.02]	[−0.05; −0.00]	[−0.03; 0.04]
Democrat received Blindspot	−0.07*	−0.01	0.01	−0.00
	[−0.10; −0.03]	[−0.04; 0.02]	[−0.01; 0.03]	[−0.04; 0.03]
Democrat received in-partisan	0.01	0.01	−0.00	−0.02
	[−0.02; 0.04]	[−0.01; 0.03]	[−0.01; 0.01]	[−0.05; 0.01]
Independent received story	−0.00	0.02*	0.01	−0.02
	[−0.02; 0.02]	[0.00; 0.04]	[−0.01; 0.02]	[−0.04; 0.01]
Non-U.S. respondent received	−0.00	0.01	−0.00	0.01
	[−0.03; 0.02]	[−0.01; 0.03]	[−0.02; 0.01]	[−0.01; 0.04]
Republican received Blindspot	0.04	0.02	0.01	−0.02
	[−0.00; 0.09]	[−0.01; 0.05]	[−0.02; 0.04]	[−0.06; 0.03]
Republican received in-partisan	−0.01	−0.03*	0.02	−0.02
	[−0.05; 0.03]	[−0.06; −0.00]	[−0.00; 0.05]	[−0.06; 0.03]
Age	0.00	−0.00	0.00	0.00
	[−0.00; 0.00]	[−0.00; 0.00]	[−0.00; 0.00]	[−0.00; 0.00]
Female	−0.01	0.00	0.00	−0.01
	[−0.03; 0.01]	[−0.01; 0.02]	[−0.01; 0.01]	[−0.03; 0.01]
White	0.01	0.01	0.02	0.01
	[−0.03; 0.04]	[−0.01; 0.03]	[−0.00; 0.04]	[−0.03; 0.04]
Black	−0.02	0.00	0.05*	0.04
	[−0.08; 0.04]	[−0.04; 0.05]	[0.00; 0.09]	[−0.04; 0.12]
Hispanic	0.01	−0.00	0.02	0.05
	[−0.04; 0.05]	[−0.03; 0.03]	[−0.01; 0.05]	[−0.00; 0.10]
Asian	0.01	−0.01	0.03*	−0.02
	[−0.04; 0.06]	[−0.04; 0.03]	[0.00; 0.07]	[−0.07; 0.02]
R^2	0.01	0.00	0.00	0.00
Adj. R^2	0.00	0.00	0.00	0.00
Num. obs.	4909	4922	4923	4909
RMSE	0.28	0.22	0.17	0.29
N Clusters	1230	1231	1231	1230

* Null hypothesis value outside the confidence interval.
