# Supplementary material for: Beyond partisan filters: Can underreported news reduce issue polarization?
Source: PLoS One. 2024 Feb 16;19(2):e0297808. doi: 10.1371/journal.pone.0297808 (PMC10871475; doi:10.1371/journal.pone.0297808)
Supplement: Attachment — Submitted filename: ReviewerMemo.docx [file pone.0297808.s002.docx]

Dear Editors and Reviewers,

Thank you for the opportunity to revise and resubmit my manuscript “Beyond Partisan Filters: Can Underreported News Reduce Issue Polarization?”

I thank the two reviewers for their extremely insightful and constructive comments. The changes I have made based on this helpful feedback have significantly improved the manuscript. I have rewritten the introduction, clarified the research design, improved the presentation of empirical results, and thoroughly proofread the manuscript.

In this memo, I reply in detail to all reviewer comments, describing where and how I incorporated revisions into the manuscript. I have responded to all concerns, suggestions, and issues raised by reviewers and editors. I include excerpts from the reviewer’s comments in this memo for clarity.

Please note that as per the resubmission guidelines, I have now included a data availability statement and have uploaded all replication data and code to the Harvard Dataverse: <https://doi.org/10.7910/DVN/QLF6MV>.

I have also updated the competing interests statement to ensure that readers have a full understanding of my partnership with Ground News, it now reads:

I worked with Ground News to implement this experiment. To minimize the appearance of a conflict of interest, I never received compensation from the company. When I presented this work together with Ground News employees at the Online News Association's 2022 conference, funding for travel was provided by Duke University; the conference registration fee was covered by Ground News. Finally, Ground News provided data on whether study participants opened the newsletter, but never had access to individual survey responses. Ground News was not involved in the analysis of these data, but has had the chance to review this manuscript to ensure that their products and business are accurately described. The company did not ask for any changes after reviewing the paper.

Finally, before turning to the substantive feedback, I have also updated my financial disclosure as I received a grant from the IHS to fund the publication fee associated with this article, should it go on to be accepted.

Starting with the first reviewer’s suggested changes, which are numbered and followed by my response (lettered):

1. “The introduction would benefit from a description of polarisation and how it relates to traditional and social media. I suggest looking at the works of Quattrociocchi, Etta, Cinelli, Lazer, De Domenico”
   a. I completely agree that the introduction did not do enough to discuss the distinction between traditional and social media and how this study fits in. I have largely rewritten the introduction, discussion, and conclusion, building on the helpful work suggested by this reviewer. I now discuss recent work on echo chambers in social media, how that work relates to past research on selective exposure, and where my study fits in.
2. “The newsletter's algorithm needs more technicalities, and, at the same time, no previous studies cited used such a newsletter.”
   a. I worked with Ground News’s editors to choose stories that were included in the newsletter for this study. Chosen stories were intended to represent the most salient news items the week the study was run. Because stories had to be selected only a few hours before the study could be run, the choices were ultimately based on the researcher’s understanding of political issues that were salient at that time. I now discuss this extensively in the paper and thank this reviewer for pointing out that I was not clear about this in the original version.
3. “The lack of this information makes the experiment lacking soundness. Please, provide a better explanation of how this algorithm works and, if possible, include other studies that have already used this newsletter”
   a. No algorithm was used in the selection of stories for the week and to the best of my knowledge no other studies have used this newsletter. The intention of using the newsletter was not so much to learn about an algorithm or the newsletter per se, but instead to learn how providing information about potentially underreported stories affected issue positions and polarization. The stories were among those covered almost exclusively by one partisan side. As I stated above, the selection of stories was ultimately based on my judgment about issue salience at the time, a point which I am now clearer about in the paper.
4. “Is bias rated on a discrete or continuous level? Please, provide a more detailed explanation of this rating”
   a. I have now added more detail on Ground News’s bias ratings to the paper. Please keep in mind that behind the scenes the Ground News ratings are proprietary, so I do not have access to them to report more detail in this paper. However, the result of their ratings are public information on their website.
5. "Ground News' content suggests that its subscribers have an intrinsic interest in exploring narratives beyond conventional partisan lines." is this sentence supported by their statement? Please provide a link to that”
   a. To the best of my knowledge, Ground News has not made a public statement about their readers. My statement is based on an assumption about the type of person who would choose to read Ground News. I believe that this is a reasonable assumption because of the explicit mission of Ground News and design of their products. That statement supports my conjecture that this population on which I am conducting the experiment will be especially likely to respond to information from cross-partisan media sources.
6. “For transparency purposes, the paper would benefit from a distribution of the biases of the newsletter on a broad timespan to quantitatively assess the objectiveness of the company itself”
   a. I completely agree that the paper would benefit from a distribution of the coverage of the newspaper over time, this would be especially true if my experiment were carried out over a longer time scale. But unfortunately, as Ground News is a private company, such a distribution is not available. I believe that this does not have a great impact on my paper, because my experiment is only conducted over a single week and I have complete control over the stories in that week’s newsletter and the surveys administered. Furthermore, in my statistical tests I have a control for how baseline opinion on each of my four issues was changing over time during that week.
7. "Consequently, they could be more open to persuasion and exhibit more flexibility in their attitudes and beliefs than others." The lack of cited studies makes this sentence just an opinion from the author. I suggest bringing studies evidencing how the lack of a political leaning leads to better persuasion. Furthermore, the part "might exhibit more flexibility in their attitudes and beliefs than others." is too general. What does having a flexible attitude or belief mean? Furthermore, please provide studies that compare those with a "flexible attitude and belief" to those who don't have that”
   a. I have added a new sentence to the start of this paragraph emphasizing that there is no way to definitively compare Ground News’s subscribers to the population. For the reasons I describe in (5a) above, I believe that my conjecture is reasonable in this context and I now make it explicit that this is ultimately a conjecture.
8. "I selected four breaking stories from July 14th 2021 to July 18th 2021 that received limited coverage from either conservative or liberal-leaning media." What does limited mean? Is there a coverage threshold or measure that defines a story as preferred from a specific political side from a more objective one?”
   a. In this paper when I say limited coverage I mean that these are stories which Ground News identifies as “blindspots” since they are not broadly covered from across the political spectrum. In the paper, I have rewritten this to clarify it (p. 3).
9. "Of the initial 2,372 respondents, 1,861 reported an email address matching a Blindspot Report subscriber." I do not get the math here. If subscribers to the Blindspot Report were invited to take the survey, how can only 1861 of them be subscribers? They should have been 2372. Please, provide a better description of this.”
   a. I now discuss this in the paper (on p. 4), but the explanation is that at the end of the survey respondents were asked to self report their email so that I could contact them again. This was kept separate from Ground News for privacy concerns. Some of those asked to report their email provided a different one from that which they used to subscribe to Ground News, which is why there is this difference.
10. “Was the survey composed of open questions or closed ones? In the latter's case, what were the questions' interval ranges?”
    a. All questions in the survey were closed (see the Appendix for exact questions and response options). All questions were asked on 5 point scales and coded from 0 to 1.
11. "In conclusion, these findings demonstrate that dismantling information echo chambers may, in certain contexts, reduce polarization and the gaps in issue positions between Democrats and Republicans." this sentence does not consider the concept of echo chambers. Here, the study does not account for the topology of the echo chambers. Therefore, no statement can be made about echo chambers but just the polarization itself.”
    a. I completely agree with this reviewer that I was incorrect in my use of echo chambers in that sentence and have rewritten it to reflect my focus on news stories covered from across the partisan divide.
12. This reviewer also suggested several grammatical and formatting changes which I have implemented.

Now for the second reviewers’ comments, which are again numbered and followed by my responses:

1. “The authors may wish to acknowledge that the manipulation, being a one-time, small change may not have had a large impact on such important outcomes as issue polarization.”
   a. I thank this reviewer for the helpful reference, and I now elaborate on the limitation that this experiment was a one-time, small manipulation in the discussion. As I now write, one can imagine much larger effects were people to consistently receive information about their Blindpots on one or many issues over time. I now add that even if this experiment were done, it would still be challenging to find consistent effects because of work showing that most people spend very little time following political news.
2. “As an improvement, I would suggest that the author make their hypotheses clearer — on page 7, they refer to “16 potential hypotheses under investigation,” but these are never explicitly outlined in the setup of the paper.”
   a. I completely agree that in the original submission I was unclear about what is or is not a hypothesis in this study. I had originally included all possible attitude-issue combinations, of which there are 16 because there were 16 key questions asked on the survey. In this new version on p.5, I go into much more detail than I had before about what I am measuring and what I am hoping to analyze in this study. I now introduce the idea of attitude-issue combinations, briefly discuss the experimental ATE results that I now expand on in the appendix, and move to the main analysis that pools issues to focus on the four substantive attitudes.
3. “For additional clarity, the significant p-values used in table 1-4 should be aligned with the p-values discussed in the text (and p-values <0.1 should not be highlighted, in my opinion). Also the p-values thresholds used in table 5 should be stated in the table.”
   a. I have rewritten the results section to consistently highlight the analyses that I am reporting in the main paper and flag those left for the appendix for readers. I have also stated p-value thresholds used in the table.
4. While the experiment is well-described on page 6 and figure 1 aids comprehension, the setup is sufficiently complex that a reader would benefit from an additional figure or table to clarify which participants were exposed to which treatments.
   a. I agree that the complexity of the design lends itself to a graphical presentation and have made this flow chart and now refer readers to it in the paper.
5. “The author sets themselves up to assess 16 potential hypotheses using Bonferroni-adjusted p-values. The resulting threshold is rather stringent, and so the authors report results under both the Bonferroni-adjusted criteria and traditional 0.05 criteria … I believe one way to mitigate this would be to lead with an aggregated hypothesis and analysis that better sets up the broader question this paper is trying to answer.”
   a. I thank this reviewer for pointing out the confusion in how I am setting up and presenting my analyses. I have completely rewritten the results section to first highlight why I believe that the individual story experimental analysis is valuable, but not the best way to look at the impact of this experiment, and now briefly discuss the Bonferroni adjusted results but refer readers to the Online Appendix for the presentation of tables. I have taken the reviewers suggestion to foreground what I now describe as the attitude-issue analysis and make clear that that is the main contribution and that we must dive into the individual issues after that, since just one of the four issues is driving the aggregated results.
6. Like the first reviewer, this reviewer suggested several grammatical and stylistic corrections which I have implemented and am grateful for.

I am grateful to both reviewers for their time and effort spent helping me improve the manuscript and I am happy to answer any remaining questions or to make any remaining changes.

Sincerely,

Curtis Bram

Assistant Professor

The University of Texas at Dallas
